# Ethical Challenges of Virtual Reality Technology Interventions for the Vulnerabilities of Patients With Chronic Pain: Exploration of Technician Responsibility

**DOI:** 10.2196/49237

**Published:** 2023-12-04

**Authors:** Siyu Zhou, Diane Gromala, Leyu Wang

**Affiliations:** 1 Department of Philosophy Central South University Changsha China; 2 School of Interactive Arts and Technology Simon Fraser University Surrey, BC Canada

**Keywords:** patients with chronic pain, vulnerability, virtual reality interventions, ethics, responsibility, technical developers

## Abstract

Chronic pain, a common disease, is a crucial global public health concern. Approximately 20% of the worldwide population is affected by chronic pain, which accounts for 15% to 20% of hospital visits. In Canada, approximately 7.6 million people—or 1 in 5 people—experience chronic pain. Among this population, 60% has either lost their employment or experienced a reduction in income as a result of their pain. The proportion of older people (aged ≥65 years) with chronic pain is high, comprising one-third of the total older population. In addition, the causes of chronic pain and its cures are unknown, and treatment is limited by these unknowns and the dangers of opioids. These essential factors make patients with chronic pain one of the most vulnerable populations. The use of emerging virtual reality (VR) technology as an intervention for chronic pain has consistently demonstrated early effectiveness and has been termed as a “nonpharmacological analgesic.” Nevertheless, we must remain vigilant about the potential ethical risks of VR interventions, as inappropriate VR interventions may exacerbate the vulnerabilities of patients. Currently, a central challenge for VR developers is the ambiguity of patient vulnerability and the unpredictability of ethical dilemmas. Therefore, our paper focused on the vulnerability and ethical dilemmas faced by patients with chronic pain in VR interventions. Through an experience-based, prospective ethical examination, we have identified both existing and potential new vulnerabilities and specific manifestations that patients with chronic pain may encounter in VR interventions. Our aim was to highlight the ethical risks that may be present in VR interventions. On one hand, this can help raise awareness among technology developers regarding the vulnerabilities of patients with chronic pain and mitigate technological ethical risks. In addition, it can assist technology developers in determining the priorities for VR technology interventions. These efforts collectively lay a solid foundation for the comprehensive realization of responsible VR technology interventions.

## Introduction

Ethics is a part of philosophy; it can only be clear in the context of a specific set of beliefs about what exists and how we can obtain knowledge. Virtual reality (VR), as a unique form of digital health technology, reflects a profound crisis in our concepts of knowledge and existence. Virtual worlds are fundamentally observer dependent (ontologically subjective), but they are rooted in (or made possible by) observer-independent states of a physical computer. This occurrence is distinct from approximately all other phenomena. This necessitates a fundamental rethinking of existing philosophical concepts and ethical frameworks [1]. Our traditional philosophical system is collapsing at its most central point, metaphysics, owing to the advent of VR, which reflects profound philosophical confusion [2]. Therefore, our concern regarding the ethics of VR is extremely difficult. VR has left ethics severely attacked and in a state of confusion for at least 50 years [3]. It has become evident that traditional ethical principles are inadequate to address the new challenges that VR technology presents. Therefore, ethics must generate a new dimension of responsibility to establish a new scope of responsibility. Consequently, we must contemplate the moral implications of VR technology and study new ethical principles to address them.

In *The Enigma of Health* (1996), Hans-Georg Gadamer argues that health is a way of being in the universe, that disease is a disturbance of that way of being—it symbolizes our essential vulnerability, and that “medicine is a compensation for the vulnerability of human existence” [4]. Currently, digital transformation provides the most advanced technological interventions in the health care field. This transformative process holds immense potential for mitigating the vulnerabilities faced by patients. VR technology, as one of the new digital health technologies, is also playing an increasingly significant role in the domain of chronic pain. Nevertheless, it is worth noting that the current application of VR technology is only adapted to contemporary health care development. It has not been extensively explored.

Goodin [5], in his book, *Protecting the Vulnerable: A Reanalysis of Our Social Responsibilities*, views the concept of vulnerability as a relational concept. He argues that “vulnerability implies that there is some agent (actual or metaphorical) capable of exercising some effective choice...over whether to cause or to avert threatened harm.” Goodin [5] provides further elaboration on this topic in 2 specific ways. A perspective posits that vulnerability implies that, in the face of specific threats, individuals are always vulnerable to harm from specific agents [5]. In the historical context of unequal power between health care providers and patients, the use of VR technology further exacerbates the difference of power, expertise, and resources between technology developers and patients with chronic pain [6]. These inequities provide technology developers an increased level of authority in determining choices and outcomes. On the one hand, this confers a special moral duty and accountability upon technology developers to use their expertise in aiding patients. In contrast, it also presents the temptation for them to exploit this asymmetry of power for their own benefit, which would further intensify the vulnerability of patients.

The medical journal *The Lancet* proposed the following in an editorial: “Continuous exposure to VR will impoverish those aspects of life that determine social development, interpersonal insight, and emotional judgment. Vulnerable patients should not be exposed to VR until the full extent of its likely impact can be reliably anticipated.” Currently, VR is an emerging technology and pain intervention, and some studies have demonstrated favorable preliminary effects of VR in managing chronic pain. However, any potentially powerful treatment modality applied to medicine seems likely to possess considerable capacity to induce adverse events [7]. Some ill effects may be reliably anticipated, but others could be quite unexpected [7] or even possibly man made. Without well-defined research methodologies and comprehensive ethical deliberation pertaining to VR interventions, practical constraints may arise during the development phase. This may delay the timely identification and comprehension of the potential negative consequences that VR interventions may have on patients with chronic pain [7]. Considering the significant diversity and complexity of characteristics that patients with chronic pain endure as a collective, their degree of vulnerability that accrues from multiple symptoms and outcomes can easily surpass that of other groups. To ensure safe future applications of VR to manage chronic pain, our primary focus is on the vulnerabilities of patients with chronic pain. On this basis, our intention was to prudently predict the ethical challenges that the use of VR interventions may pose to the vulnerability of patients with chronic pain. On the one hand, this contributes to eliciting moral responses from the moral agents, namely VR technology developers, thus increasing their sense of responsibility. It prompts technology developers to eliminate or at least minimize these potential negative impacts during the research and development phase of VR rather than await reports of its ill effects from VR intervention. As history demonstrates, this strategy is far more cost-effective than improving VR after its widespread clinical application. In addition, early consideration of ethics may effectively prevent the possibility of patients with chronic pain becoming even more vulnerable as a result of VR interventions and the potential harm that VR technology applications might generate. We hoped to engage in responsible reflection, design, improvement, and innovation of current VR technology by means of meticulous forecasting and scholarly investigation. The objective of this study was to explore the development standards that are specifically designed for interventions in chronic pain using VR, thereby exploring the ethical responsibilities of VR technology developers and harnessing the power granted by VR technologies in a responsible manner to achieve our ultimate goal of providing more personalized, targeted, and patient-centered care and active treatment for patients with chronic pain, while striving to maintain their dignity and personal integrity.

## VR for Chronic Pain

With the substantial development of VR, its applications have steadily expanded from the initial aviation flight simulation during World War II to various fields, such as medical health interventions. Currently, VR is applied to a variety of medical and health-related fields, such as psychology, cognitive science, and clinical medicine [8].

In 1998, Hoffman et al were the first to propose using VR technology as a tool for pain modulation [9,10]. Since then, pain, as a clinically comprehensive discipline, has benefited greatly from VR. VR has been demonstrated to act as an effective supplement or alternative to opioid analgesics for pain relief in surgical or acute pain management [11-17].

Chronic pain is recognized as a disease by the World Health Organization and is divided into chronic primary pain and chronic secondary pain [18]. It is defined as pain that persists or recurs for >3 months. Such pain often becomes the sole or predominant clinical problem in some patients [19]. Meanwhile, it is considered by some to be a complex disease or condition, even though the factors that lead to chronic pain are often unidentified and have no known biomarkers or cures [20].

Chronic pain involves multiple variables, such as neurobiological, psychological, and social factors, that are distinct from acute pain. Moreover, chronic pain, by definition, is longitudinal and requires more involved, long-term research studies. Thus, the extent to which the applications of VR are used in the intervention of chronic pain is still in its infancy. Therefore, the effect of VR on intervening chronic pain is observed more often in clinical research than in routine treatment. So far, a few studies have shown that VR intervention can dramatically alleviate chronic pain symptoms. These study data continue to support the investigation of the application of VR in the treatment of chronic pain, and VR intervention seems promising as a nonopioid therapeutic modality for chronic pain and to benefit patients with chronic pain [21,22]. Before exploring the ethical challenges arising from the vulnerability of VR interventions for patients with chronic pain, sorting out the methods and mechanisms of VR technology interventions for chronic pain will facilitate a more appropriate and in-depth reflection about and improvement of VR technology.

In the 1980s, Jaron Lanier founded the Virtual Programming Languages company and coined the term *VR* by commercializing the first display products capable of displaying a computer-generated alternative reality. The VR paradigm has evolved from the initial stage of projecting computer graphics on large displays to Cave Automatic Virtual Environment–like systems with 360 dfs to more recent technologies using high-resolution head-mounted displays (HMDs) [23]. It is important to clarify that the VR discussed in this paper refers to immersive VR.

Immersive VR technology typically consists of an HMD with head tracking; headphones with sound or music and noise reduction; and a joystick, vibration pad, or other device for manipulation and navigation [24]. It designs interactive VR environments using 3D computer graphics projected by VR HMDs [25,26].

Owing to competing theoretical explanations for pain, the precise mechanisms by which VR may alleviate it are uncertain [27-35]. Theoretically, VR analgesia results from the neurobiological interactions of brain regions that produce analgesic effects by modulating visual, auditory, and tactile sensory experiences [32]. Currently, VR predominantly relieves chronic pain by competing for the patient’s attention [24]. McCaul and Malott [31] note that humans have a limited capacity for attention and that a painful stimulus must be attended to, to be perceived as painful. Therefore, if a person is attending to a stimulus other than the excruciating stimulus, they will perceive the painful stimulus as less intense. According to the multiple resource theory by Wicken [36], distinct sensory systems’ resources operate independently. This supports the nature of VR technology, which is based on integrating multimodal (visual, auditory, tactile, and olfactory) sensory distractions [24]. By placing patients in immersive virtual environments (VEs) that occupy their finite attentional resources and compete for their attention that should be directed to the painful stimulus, external stimuli related to the real environment and the painful stimulus are blocked to influence their pain perception [37]. The diversion of attention by VR can be achieved by either directing inward or outward.

**Figure 1:**
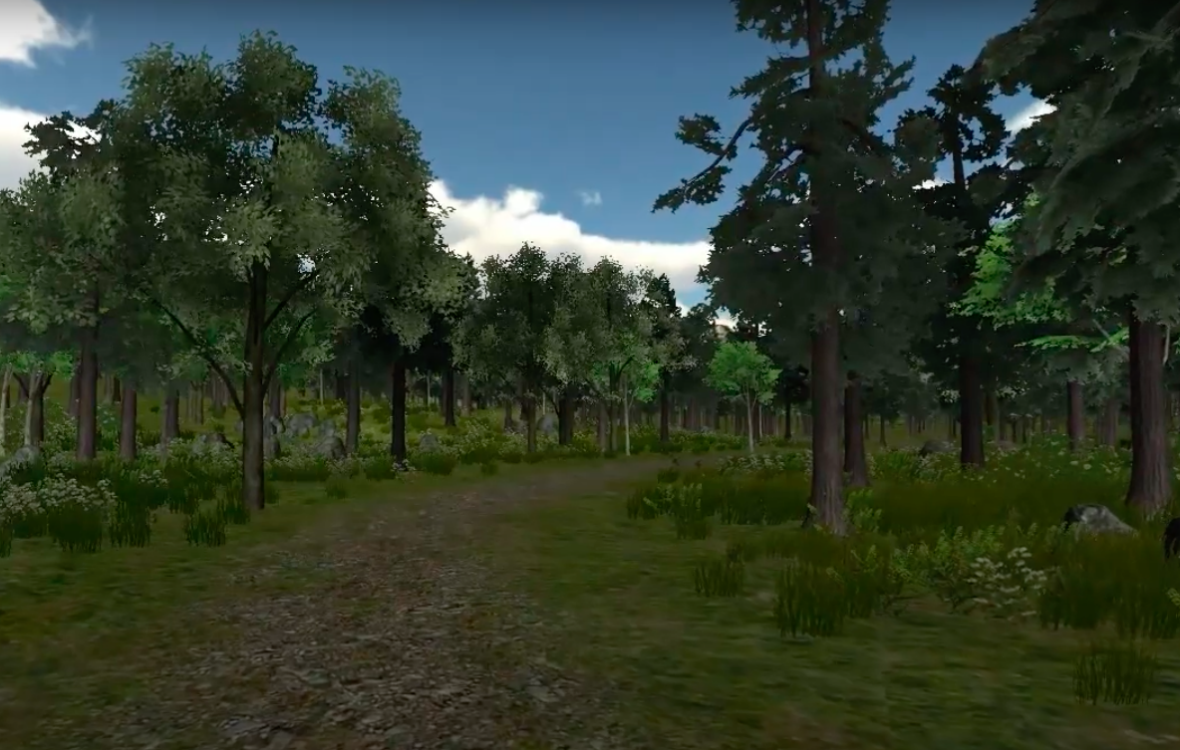
Virtual environment—Virtual Meditative Walk. Patient walking through the forest.

**Figure 2:**
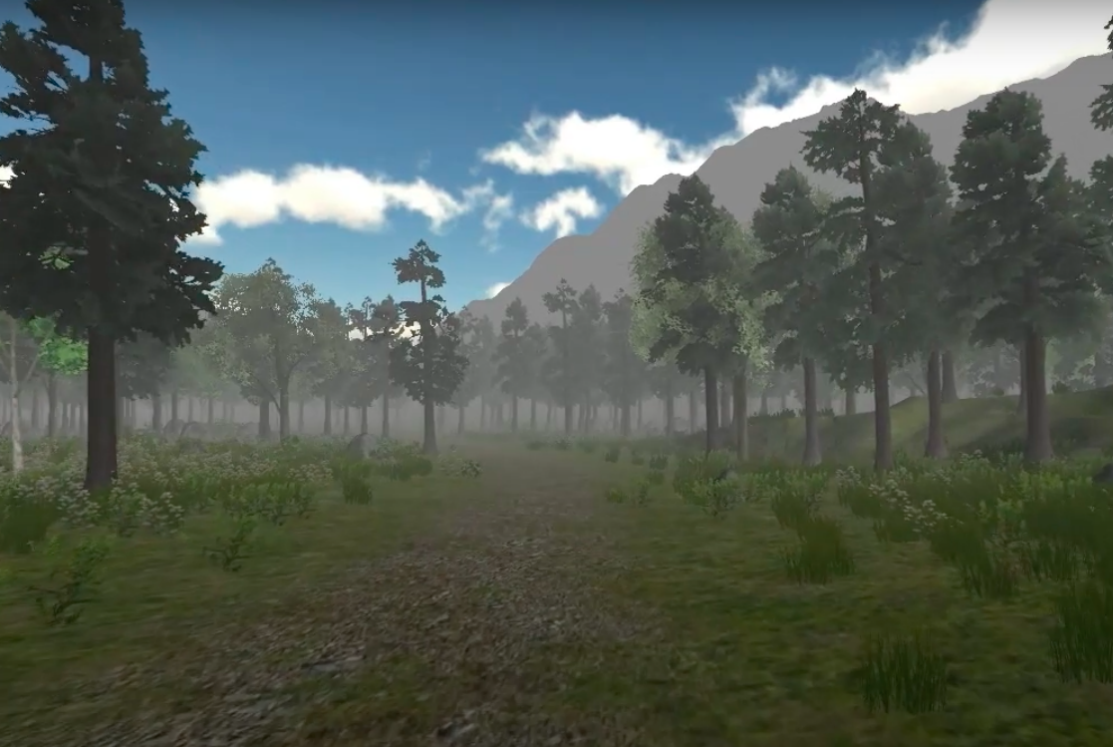
Virtual environment—Virtual Meditative Walk. As patients become more relaxed and immersed, the virtual environment will exhibit an increased presence of fog.

Directing attention inward primarily focuses on using VR environments for mindfulness meditation practices [38-40]. Mindfulness meditation is a nonpharmacological method for managing chronic pain. Its primary objective is to improve the maintenance of the patient’s mental state by reducing stress and improving health by focusing on the patient’s internal state [41]. It has been demonstrated that mindfulness meditation can effectively regulate pain sensations [42,43]. Given this idea, researchers have attempted to combine VR with mindfulness meditation. Gromala et al [44,45] were among the earliest researchers to explore the use of VR environments for mindfulness meditation in patients with chronic pain [46]. They designed the Virtual Meditative Walk (Figures 1 and 2) VR application [45]. Once patients enter the VE, galvanic skin response sensors continuously track their changing arousal levels. On the basis of the patients’ biofeedback data, the VE is modified to assist them in achieving a better state of mindfulness, thereby alleviating their pain [45]. Their controlled experiment on 13 patients with chronic pain revealed that the combination of VR, mindfulness meditation training, and biofeedback reduced reported pain levels significantly more effectively than mindfulness meditation alone [45].

Directing attention outward is primarily accomplished through interactive VR environments such as VR gaming and VR exploration. The application Snow World as an intervention for patients with acute burn has been used in almost all VR studies. Cool, a product developed by DeepStream VR that includes some engineers who worked on Snow World, is one of the earliest studies to apply VR to chronic pain interventions [47]. In the Cool VE, patients can experience the changing seasons and play with the creatures in it. During the use of Cool in VR interventions, patients experienced 60% to 70% reduction in pain, and the analgesic effects persisted for up to 48 hours after the intervention. The study results support the broad use and further investigation of VR pain control therapy as an adjunct to or potential replacement for pain therapy [48]. Building upon the foundation of using patient distraction to alleviate chronic pain, interactive VR environments can further indirectly alleviate chronic pain through the design of physical rehabilitation. For instance, the immersive VR game Lumapath, developed by Tong et al [49], includes VR game tasks based on movements inspired by tai chi, yoga, or Pilates. According to experimental findings, Lumapath immerses older patients with arthritis, distracting them from their ongoing physical activities and stimulating their participation in physical movement [49]. In addition, several researchers have explored the use of VR for treating phantom limb pain [50-53]. By using VR, clinicians can present patients with a virtual representation of their missing limbs. Patients experience relief from phantom limb pain as a result of perceptual and motor training in which their virtual limbs move in response to their voluntary motion signals [40].

## Ethical Challenges of VR Technology Interventions for the Vulnerabilities of Patients With Chronic Pain

### Overview

As early as the Hippocratic oath, it was expressly stated that health care professionals have an obligation to fulfill the obligations of nonmaleficence and beneficence. Respect for autonomy, nonmaleficence, beneficence, and justice are currently the 4 principles of biomedical ethics. The principle of nonmaleficence obligates health care workers to abstain from causing harm to others. When combined with the beneficence principle, it generates four norms [54]:

1. One ought not to inflict evil or harm (nonmaleficence)
2. One ought to prevent evil or harm (beneficence)
3. One ought to remove evil or harm (beneficence)
4. One ought to do or promote good (beneficence)

As a digital health technology for intervening in chronic pain, VR should therefore adhere to the principles of biomedical ethics. This involves not only preventing intentional harm with VR but also encouraging proactive measures by technology developers to prevent harm, remove harm, and benefit patients. As patients with chronic pain have high vulnerabilities, they are susceptible to potentially unexpected adverse effects if the developers of the technology and applications are unaware of the experiences common to these patients, such as catastrophizing or kinesiophobia, resulting in inappropriate or unsafe VR interventions. This plainly violates the nonmaleficence and beneficence principles. Before exposing patients to VEs, we must therefore carefully consider whether VR interventions may exacerbate patients’ vulnerabilities. This will help VR developers to better and more precisely avoid the possible risks associated with VR technology for this large demographic.

### Inherent Vulnerabilities of Patients With Chronic Pain: VR Immersion

#### Overview

Several extensive population-based surveys demonstrate that 1 in 5 Canadians experience chronic pain [55-57]. Overall, two-thirds of Canadians with chronic pain report that their pain is moderate (52%) to severe (14%), and 50% have experienced chronic pain for >10 years [56]. Simultaneously, the prevalence of chronic pain continuously rises as adults age, making this a serious health problem for older people, who are increasing in number. Chronic pain affects approximately one-third of Canadians aged ≥65 years [56,57]. According to the abovementioned statistics, the community of patients with chronic pain has integrated features, including high patient count, aging population, intense pain, and lengthy illness term. Furthermore, the disease complexity and incurability of chronic pain are reflected in ill-health effects that may lead to anxiety, depression, insomnia, decreased mobility, and increased social isolation among patients with chronic pain. A World Health Organization study, for instance, indicated that those with chronic pain are 4 times more likely than those without pain to experience depression or anxiety [58]. These symptoms negatively affect the ability to work, ability to function in day-to-day tasks, and quality of life of patients with chronic pain. If individuals with persistent pain have other underlying disorders, the overlap of multiple diseases further increases their risks. Moreover, chronic pain is one of the top 10 causes of disability [59], and up to 10% of individuals experience severe disabling chronic pain [60]. All these features are inherent in the state of patients with chronic pain and stem from the patient’s corporeality, needs, reliance on others, and emotional and social milieu [61]. We refer to these characteristics as the “inherent vulnerability” of the population of patients with chronic pain. Although varied and complex, these inherent vulnerabilities are one of the reasons why the population with chronic pain is more vulnerable than groups termed as “healthy population” or other populations defined by a specific disease.

Immersion is one of the primary characteristics of VR, influencing a person’s sense of place and time or perception about the world [62]. VR interventions for patients with chronic pain rely primarily on facilitating their immersion in VR to distract or induce analgesic effects, learn rehabilitative skills, or maintain physical activity. However, high degrees of immersion and long-term immersion may expose patients who are already vulnerable to significant potential damage. Therefore, this section will concentrate on discussing the ethical challenges these 2 states of immersion may pose regarding the inherent vulnerability of patients with chronic pain.

#### High Degrees of Immersion

Pain requires attentiveness, and humans have been found to have limited controlled attentional resources [41]. The level and impact of distraction can depend on the level of immersion—the more immersive the VR, the more effective it is in reducing pain [63]. Although distraction has not proven that the analgesic effects of VR persist beyond a VR session, distraction can be important for unexpected “break through pain,” and just being able to access short-term relief in itself can be valuable. More long-term VR interventions intend to “retrain the brains” and help maintain the range of motion [49,64]. Although not all VEs have the immersive aspects tested, it is widely held by VR developers that perspectival fidelity or context realism of VR content creates an “almost real experience,” thereby promoting a high degree of immersion.

In terms of technology, perspectival fidelity refers to the degree to which a representation accurately depicts the subjective point of view of a neurotypical human being. Representations that are highly faithful to human perspectives are considered more likely to generate more immersive VR experiences than less faithful representations [65]. Perspectival fidelity is primarily adjusted through the design and adjustment of the virtual experience structure, such as adjusting the relative height of the virtual avatar, making the depth of field visible in the virtual landscape, and designing the virtual experience from a first-person or third-person perspective. In addition, hardware elements such as the weight of the VR headset [66] and the refresh rates of graphics [67] can also influence the perspectival fidelity of the experience.

In terms of “content”—or what the patient sees, hears, feels, and interacts with—context realism focuses on the degree of realism that VR content provides to patients, primarily in terms of the plausibility of the virtual surroundings [65]. The perception about realism is enhanced when the visual and audio elements conform to the laws of the physical world, such as when patients observe virtual objects obeying the existing physical laws of mechanics.

To increase the sense of immersion, VR technology developers exert significant effort to reconstruct a digitally simulated world that closely resembles reality, with the goal of minimizing the distinction between the real and virtual worlds [68]. Theoretically, this could diminish the patients’ psychological ability to distinguish between the real and virtual worlds [69]. Unfortunately, the efforts of VR developers to provide highly immersive experiences by emphasizing photorealistic simulations that are approximately identical to the real world may result in actual damage to the patients in several ways.

Highly vivid VEs emit a vast quantity of information in the visual, aural, tactile, or even olfactory modalities. The corresponding output devices are frequently very obtrusive, in that they are directly attached to or very near the patient’s senses during the reception process. For instance, headphones almost inevitably project sound into the patient’s ears. It is nearly impossible to shut down that sensory channel by consciously trying to filter out auditory information. The same holds true for visuals. In HMDs, patients have stereoscopic displays in front of their eyes to receive visual stimuli; the HMDs simultaneously occlude vision of the real world. Handheld controllers often also emit vibrations that function as a form of feedback. The amount of this pervasive, multisensory information may cause the problem of information overload [70], particularly when the VEs are overtly designed as a form of video game, which is often characterized by fast-moving, highly stimulating game conventions. Given the inherent vulnerability of patients with chronic pain compared with healthy individuals—particularly during breakthrough pain flare-ups, they have an extremely limited capacity to process information. Moreover, when patients are exposed to an excessive amount of information that exceeds their pain-impaired coping abilities, sensory overstimulation may result in negative outcomes such as stress, depression, feelings of frustration and disillusionment, health problems, impaired judgment, and decision-making errors [71] and may trigger kinesiophobia or catastrophizing.

The VE that controls the VR simulations is determined by designers using software applications, in other words, by programmers rather than by nature [7]. This implies that the closer the simulation is to reality, the more complex the programming and design and the greater the likelihood of errors. For instance, compared with the natural world they simulate, complex VEs may have deficiencies in fidelity, quantity, consistency, and responsiveness of the diverse physical stimuli that are present in natural experiences [70]. In addition, owing to the complexity of chronic pain and the competing theories regarding pain, there are currently no research teams that have proposed specific VE standards for VR interventions in chronic pain. This absence of standards may lead to the implicit inappropriateness or explicit abuse of VEs. When patients with chronic pain immerse themselves in VEs and are confronted with limited, misaligned, or incoherent sensory inputs and the possibility of VE misuse, they may experience perplexity; frustration; impairment [70]; or triggering of their hypersensitivity to sound [72], visuals, or haptic output. The most prominent phenomenon in such situations is the commonly known “simulation sickness” or motion sickness [73-76]. In general, the use of mixed signals to confuse the signal processing of the brain can readily induce extreme motion sickness [77], and complex VEs may negatively affect how fast the computer can display real-time changes in what a patient sees, for example, when they simply turn their heads. Even if patients promptly exit the VR intervention by removing the VR headset, symptoms such as nausea, vomiting, cold sweating, pallor, salivation, drowsiness, dizziness, headache, eye strain, lethargy, lack of initiative, and chronic fatigue may persist for an extended period [74]. In rare cases, the onset of symptoms may be delayed. In addition, using VEs to alleviate pain for patients undergoing chemotherapy requires special consideration. When patients undergoing chemotherapy are highly immersed in a realistic VE, mismatches between the graphics and the patient’s movement speed are more likely to induce motion sickness. Moreover, when patients are highly immersed in a VE and fail to detect distortions, they may become powerless or unable to timely counter the potential risks of outcomes such as sim sickness. For example, through programming VR or designing VEs, technology developers may introduce specific distortions into the mental lives of susceptible individuals [7]. This could exacerbate psychological symptoms, such as depression and feelings of isolation or loss of agency that already resulted from their chronic pain [78], or even provoke mental health symptoms that had never been experienced.

Apart from potential psychological harm, studies indicate that in situations where the types and intensity of VEs that patients with chronic pain face are not explicitly specified and when patients experience highly immersive VEs, it can easily trigger negative emotions similar to real experiences and that those negative emotions can be heightened by the highly immersive VR intervention [70]. Therefore, patients may exhibit negative physiological and behavioral responses that are consistent with their actual experiences [79-81]. Furthermore, when potentially unsafe elements are present in the VE, there is a substantial risk for patients with chronic pain. For instance, photic stimulation–induced seizures are an absolute contraindication [82]. In addition, sharp objects or high-pitched noises have the potential to make patients with chronic pain more irritable or cause them to be susceptible to mental illnesses. As chronic pain inherently has biopsychosocial aspects by definition, these stimuli may exacerbate their pain symptoms or induce other disease manifestations, thereby intensifying the inherent vulnerabilities of patients with chronic pain.

In addition, the real harms associated with high degrees of immersion include the potential harms of the physical environment. In terms of vision, for example, the essence of the design of the HMD is to cut off or occlude the patient from seeing the real world [83]; isolate the patient’s visual senses; and direct them almost exclusively to the stereoscopic, immersive VE. However, this renders the patient less capable of physically detecting encircling events in the physical world they sit or stand in, which is a potential and well-known physical danger [84]. Similarly, using headphones to listen to the music and audio aspects of a VE can further enhance the patient’s sense of immersion. Here also, the headphones isolate the patient’s auditory perception from the outside world. Audio from the physical world that may be detected can additionally be eliminated with noise-cancelling software and hardware such as closed-back, in-ear headphones [85]. The predominant approach of VR developers is to design VR hardware, software, and content to isolate a patient’s visual and auditory senses from the real world precisely to increase their sense of immersion in the VE. This causes patients to lose awareness of the real world, which may hinder their ability to detect and respond to the common risks of bumping into or tripping over physical objects, pets, or children. Directing a patient’s senses and attention completely to a VE also bears the risk of being less able to respond to less common incidents such as fires or natural disasters in a timely manner. Even though using VR in familiar environments can significantly mitigate this real harm, the physical environmental risks are still present and should not be ignored.

A high degree of immersion in an almost-real VE increases the likelihood of real harm to patients with chronic pain, which conflicts with the principles of nonmaleficence and beneficence. Most of the potential harm may arise from the developers’ pursuit of a more realistic and highly immersive experience, which we know is intended for the benefit of patients. To avoid exacerbating the inherent vulnerability of patients with chronic pain, however, developers also have a responsibility to proactively prevent these issues during the VR development phases. This includes adjusting or reducing the perspectival fidelity of the VE and the context realism attributes based on the patient’s specific conditions.

#### Long-Term Immersion

The Canadian Pain Society Task Force considers waiting for >6 months for pain care to be medically unacceptable [86]. In reality, however, more than one-third of publicly financed pain clinics in Canada have wait periods exceeding 1 year, and most of the regions lack access to adequate pain care [87]. This not only causes patients to experience deteriorating health, such as increased pain and depression [88], but also exacerbates their financial burden and, more gravely, can directly result in mortality. A study published in the *Journal of the Canadian Medical Association* further exposes the tragic reality that most of the deceased had visited a physician (emergency room or office visits) within 9 to 11 days before death and obtained a mental health or pain-related diagnosis. In approximately one-fourth of the cases, the medical examiner found that suicide was the cause of death [89]. Eventually, these patients did not receive the necessary assistance they needed [90]. VR interventions that have been clinically tested may serve a potentially important role during this extended waiting period. However, currently, VR is an emerging intervention for chronic pain management and is primarily in the clinical research stage; thus, access to VR interventions in a clinic or home is low because of cost, technical complexity, and lack of adoption in highly regulated clinical contexts. For instance, the cost of a complete VR system that operates a high-quality VR clinical experience is approximately US $2500 per unit, in addition to equipment maintenance expenses, resulting in expensive clinical research costs for multiple patients [40]. VR systems that use a patient’s smartphone are significantly less expensive and thus more accessible for use in a patient’s home, but these have significant limitations and still require complex upgrades and maintenance.

Besides the extended wait times for chronic pain treatment, patients dealing with chronic pain, particularly those who are disabled by it, face challenges related to limited mobility and the cost-prohibitive nature of transportation to hospitals or clinics. However, VR interventions are quickly gaining widespread acceptance and are being increasingly incorporated into clinical practices. It is expected that in the near future, business models and economic incentives will be developed to drive great accessibility, alongside advancements of more innovative, affordable VR devices and a trend toward making VR technology more domesticated and personalized. In these ways, the phenomenon of long-term immersion in VR experiences will become increasingly possible. So far, scientific research involving the use of VR has been subject to stringent control by experimental researchers or clinical caregivers, limiting immersion periods to minutes rather than hours, and long-term studies of more frequent use of VR in homes are still rare. Consequently, we are oblivious to the consequences of long-term immersion. Once VR technology becomes available for personal or domestic use, there will be no limits on the time patients choose to spend immersed [91]. To ensure adequate preparation, we need to extensively explore the ethical challenges posed by long-term VR immersion for patients’ vulnerabilities.

VR addiction is one of the primary ethical risks of long-term immersion. According to the definition of addiction, it is a compulsive and harmful desire to engage in or perform something regularly [92]. Increased social isolation among patients with chronic pain is attributed to factors such as fatigue and decreasing mobility, which are correlated with high rates of early morbidity. Moreover, the “invisibility” of pain and the lack of public knowledge about chronic pain are associated with its considerable social stigma. Crucially, pain has a tendency to “render patients without language” and even actively destroys language, ensuring that pain is not shareable through articulation in language [93]. This, combined with the public’s general lack of knowledge about chronic pain as a “real” condition, leads to social stigma.

In contrast to the uncontrollable aspects of the real world, the variables in a VE are highly controllable, with technical developers determining the patients’ experiences in VEs. VEs, unlike physical ones, can be modified rapidly [91]. Designers and programmers can create an imaginary world that suits the patient’s desires [94] and a clinician’s therapeutic goals. This may hold considerable appeal for patients with chronic pain who experience mobility limitations, social stigma, and social isolation. Such persistent burdens may lead some patients to develop a preference for the virtual world over time. However, this may further enhance the patient’s sense of isolation after exiting the VE [84]. Moreover, VEs may be significantly preferable to the actual world, particularly for patients whose quality of life has been considerably diminished by chronic pain or pain-related disabilities. It is argued that dependence on VEs may be just as easily inducible in patients as dependence on opioid medications [7]. Science fiction writers and filmmakers anticipated such problems in their early depictions of VR in popular culture. Critics such as Nozick [95] anticipated the VR in his discussion of the “experience machine,” in which he stated that “plugging into an experience machine limits us to a man-made reality.” In addition, his critique raises fundamental ethical issues surrounding the introduction of patients to VR. These concerns include free will, the nature of interpersonal relationships, and how we comprehend the repercussions of our interpersonal conduct [7]. These considerations arise on the premise that patients will be immersed in a VR environment for extended periods. After the domestication or personalization of VR technology, the social isolation of patients with chronic pain or the desire of patients with physical pain to use VR interventions for an extended period for pain relief could result in overuse of VR. This drastically raises the likelihood of VR addiction. As predicted by Nozick [95], the experience machine might reduce patients’ reliance on medication, but it also possesses a similar capability to induce or at least evoke another form of dependency [7]. Furthermore, the availability of and enthusiasm generated by the rapid development of VEs for entertainment purposes will enhance the viability of expanding its applications to medicine [7]. Nevertheless, taking into account the habits of different industries, we should reflect about the presence of violence and torture in video games. Many VEs used in health care 5 to 10 years ago were first-person shooter games. This is thought to be the consequence of the widespread use and accessibility of VR for video games. Given the expense of developing “bespoke VE” designed specifically for therapeutic purposes, reusing a commonly available VR game was rarely questioned. However, as such bespoke VEs are increasingly being developed for health care, the designers of such 3D world often come from video game backgrounds. Therefore, video games may have subconsciously and invisibly transferred the conventions and values of video games to their design practices in the medical field. However, many VR developers point out that these domains differ more often than not. Certain antisocial and aggressive behaviors are more likely to occur if susceptible patients engage in long-term immersion in VR activities characterized by peculiar or destructive fantasies without any restraint [7]. People with chronic pain detest the word *game* because they believe it trivializes their actual experience and reduces it to “play.” This is only to increase the social stigma these patients face and denigrate how serious persistent pain and its degenerative, disabling effects really are. Frequently, video games are structured in a “level-based” manner, where advancement to the next level is contingent on the completion of the current assignment. If patients with chronic pain were to engage in long-term immersion in a VE that has been designed by technology developers as an endless “level-based game,” it could fail to provide the brain calm they desire and, alternatively, cause emotional instability. This risk is exacerbated when patients repeatedly fail to progress through these levels, easily inciting wrath and potentially violent tendencies. Through these potential psychological harms, VR addiction exacerbates the inherent vulnerabilities of patients with chronic pain.

Apart from VR addiction, the ethical risks of long-term immersion could potentially lead to damage to the neural mechanisms responsible for creating the perception about reality, termed as derealization syndrome [96]. This disorder can be characterized as having chronic feelings or sensations of unreality [91], making it difficult for patients to distinguish between the real world and VE. Patients may exhibit varying degrees of symptoms, ranging from mild fatigue, daydreaming, or headaches to more severe manifestations, such as genuine chronic dissociative disorders [97]. Patients exposed to highly immersive VR environments often exhibit a certain degree of familiarity with the system’s content and adapt to its perceptual and physical parameters [98]. If patients are immersed for an extended period in highly authentic VEs, they gradually become accustomed to the VE. Moreover, heavy VR users may begin to experience the real world and their real bodies as unreal after leaving VE or effectively transfer their perception about reality to the VE [91]. This could hinder the patients’ readaptation to reality and the physical and social parameters associated with it. Some patients with derealization syndrome report a sense of being automata (loss of agency) and living in a dream-like state [91]. A small-scale study conducted with 30 participants revealed that by using different scales to quantify the derealization symptoms, preexisting dissociative symptoms increase their intensity in the VE, but the time of exposure may play an important role in their appearance [99]. Patients with depression or anxiety are more susceptible to experiencing such symptoms [100]. Therefore, we anticipate that prolonged immersion in VR could potentially lead to neurophysiological damage in patients with chronic pain. This could exacerbate patient discomfort or trigger new disease manifestations, presenting a potential risk of enhancing inherent patient vulnerabilities.

### Cognitive Vulnerabilities of Patients With Chronic Pain: VR Manipulation

#### Overview

Health care experts provide numerous beneficial methods, such as disease explanation, to help patients recognize chronic pain from the beginning of the condition. However, as chronic pain is a long-term disease, patients with chronic pain may be better able to recognize the uniqueness of their disease and its impact on their lives than physicians, as they pay long-term personal attention to their sickness, the unique presentation of their disease, and often necessary self-care or self-management. The health care experts’ prognosis of an individual patient’s disease prospect is mainly dependent on statistical reference laws based on previous clinical data and their experiential medical expertise, rather than being fully established on the disease manifestation and changes specific to the individual patient. This forecast exceeds the boundaries of medical knowledge and becomes an evaluation and reasoning problem; therefore, even health care professionals with expertise and experience are helpless to provide answers [101]. In addition, because health care workers may have potential biases toward certain patient groups, such as race and sex, they may not always be able to consistently provide sufficient assistance to the patient as their chronic pain changes over time. Therefore, when making predictive judgments for diagnosis and treatment, what distinguishes patients with chronic pain from conventional disease diagnoses is their more dominant cognitive awareness of their individual condition.

Currently, the use of rising VR technologies is progressively becoming used within the realm of chronic pain. VR can immerse patients in VEs or create certain illusions by delivering a false sense of agency, all of which contribute to the analgesic effects of VR interventions. Simultaneously, this phenomenon presents technology developers with the opportunity to manipulate the cognitive perceptions of those experiencing chronic pain through VR technology, potentially eroding the distinction between the virtual world and reality for these patients. These cognitive changes may additionally result in shifts in the emotional and behavioral responses of patients. Hence, it is imperative to maintain a state of alertness regarding the potential hazards linked to the manipulation of VR technology and explore how VR technology manipulates patients to regulate the behavior of technology developers. This enables technology developers to intervene by means of VR manipulation within reasonable boundaries, thus avoiding the erosion of patients’ original cognitive advantages resulting from the abuse of VR manipulation and ensuring the safety of patients.

#### False Sense of Agency

In experimental studies with patients with chronic pain, the visual feedback of an “embodied” dummy or virtual body in VR has been shown to effectively modulate pain sensations [41,102-104]. To generate a strong illusion of ownership for the virtual body in patients, it is imperative for VR technology to accurately monitor the self-generated motions of the patient’s physical body and synchronize the movements of the virtual body accordingly [91]. When the system operates effectively, patients perceive an illusion of ownership and agency over the virtual body, leading them to believe that they possess control over it and that it belongs to them. In actuality, the manipulation of the avatar is consistently mediated by technicians [91]. The manipulation of patients’ sense of agency can be readily achieved by technicians through the creation of a false sense of agency through avatar movements that do not align with the patient’s actual bodily movements [91]. We can gain significant insights into how technicians manipulate individuals to have a false sense of agency through VR by analyzing 2 VEs related to chronic pain: the “virtual hand illusion” [105] and AS IF.

The virtual hand illusion is an extension of a VE known as the “rubber hand illusion” [106]. A fundamental distinction is the inability of a rubber hand to exist in the same physical location as the real hand, whereas a virtual hand possesses the capacity to colocate with it. This phenomenon amplifies the analgesic efficacy of VR. The virtual hand illusion induces a sense of illusory ownership over external body parts, primarily based on visuo-tactile correlations in immersive VR technology. This congruous, multisensory input can readily result in patients experiencing illusory ownership and a false sense of agency, making them feel that the virtual limb is an inherent component of their own bodily self [106]. The patient’s sense of ownership over the virtual limb is a critical factor in attaining the analgesic outcome [107]. Some studies have certainly examined the use of this phenomenon to address pathological disorders, such as chronic pain. These studies have placed particular attention on the analgesic effects of cross-modal perception, such as pain and vision [108,109]. One of the most typical examples is phantom limb pain. Individuals with phantom limb pain use this illusory visual feedback as a means to perceive the continued existence of their amputated limb [103]. Previous studies have demonstrated that inducing a perceptual illusion of reduced size in a patient’s painful limb can lead to decreased pain perception associated with that particular limb [105]. In addition, patients can observe dynamic changes in the skin color of their embodied avatar’s limb, and different colors of visual feedback can modulate the patient’s pain threshold under heat stimuli. In contrast to bluish skin color, reddish skin color is associated with low threshold [102]. It is evident that technicians possess the ability to manipulate not only the VE but also the embodied virtual body in ways that would be impossible in physical reality [110]. For example, by means of design and programming, patients can be made to see diverse virtual body representations in terms of shapes, structures, colors, and sizes [111-114]. Moreover, studies indicate that the manipulation of virtual bodily attributes may affect the physiological reactions of the patient’s real body [102,115] and possibly modulate their behavioral responses [116,117]. Technicians are using the manipulation advantages of VR technology, which have significant potential in the treatment of chronic pain.

**Figure 3:**
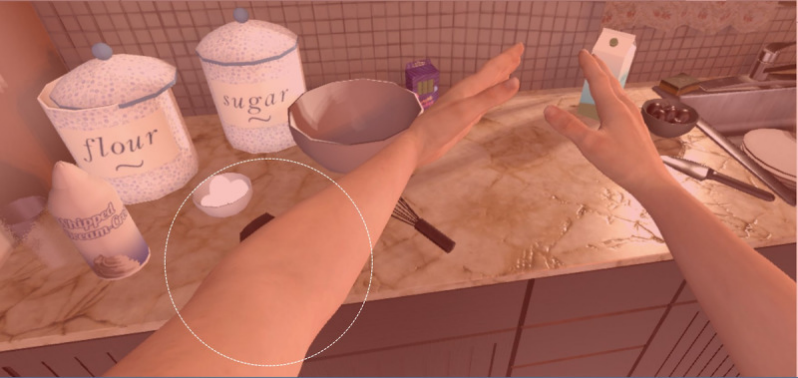
Virtual environment—AS IF. This figure shows the avatar going to get the food needed to make the cake on the table.

**Figure 4:**
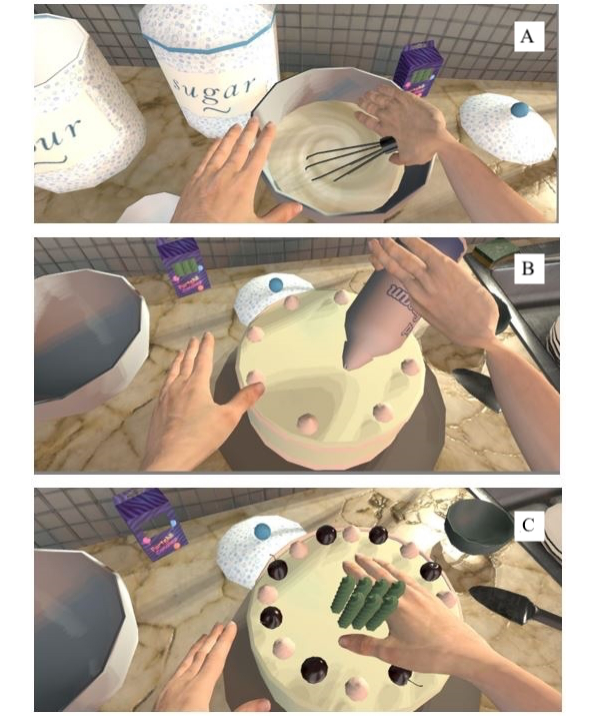
Virtual environment—AS IF. This figure depicts the cake-making process.

AS IF [118] (Figures 3 and 4) is a serious virtual game and is not intended to be used as a therapeutic intervention for chronic pain management. Its objective is to offer nonpatients an insight into the subjective experience of living with a disabled or limited body owing to chronic pain. This serves to contribute to the knowledge and comprehension of health care professionals, family members, and acquaintances with regard to those who experience chronic pain, to alleviate the feelings of isolation and societal stigma that are commonly encountered by patients. During gameplay, technicians impose limitations on the avatar’s range of motion, effectively augmenting the level of difficulty for the avatar in completing motor tasks inside the virtual game setting. The implementation of this “disabling” function facilitates the deliberate misalignment of the interactor’s actions with the avatar. Interactors may become aware of the considerable exertion involved in continually trying to get food from a table. This scenario would not occur for a healthy individual in real-world circumstances. The purpose of this VR manipulation is to allow the interactor to gain insight into the feeling of living with constraints imposed by chronic pain.

Both these VEs manipulate patients through VR control to generate a false sense of agency, subsequently affecting chronic pain from either within the patient’s body or from the environment. The cognitive abilities of patients under VR manipulation exhibit vulnerability, rendering them confronted with difficulties in discerning between the virtual and real worlds, thereby resulting in instances of experiencing illusions of ownership of the virtual body or believing that they are personally controlling the avatar to complete gaming tasks. When disregarding therapeutic intentions, the creation of a false sense of agency in VR undeniably infringes upon the user’s autonomy. Technicians have control over the virtual world, determining how patients cognize and interpret it, and patients can only receive the information that the virtual world’s designers intend them to receive. In addition, technicians possess the capability to readily modify the virtual world to cater to particular objectives that have an impact on individuals’ beliefs, emotions, and behavior. The manipulative potential can be easily used for a wide range of goals, ranging from commercial to political fields [119]. Ford [120] has critiqued VR regarding the risks of problematic representations in VEs, including inaccurate and biased representations of people and objects mimicked in VEs [121]. Misrepresentations have the potential to significantly influence users’ cognition of genuine individuals, groups of people, objects, and various views on real-world matters [119]. As pointed out by O’Brolcháin et al [122], VR programs have the potential to exert influence on users’ offline behaviors through various manipulative ways [91]. When these users are patients with chronic pain, their inherent vulnerability, in conjunction with the cognitive manipulation facilitated by VR, could potentially render them more susceptible to the negative impacts stemming from false or biased representations.

#### Commercialization of VR Technology

The influence of VR manipulation on patient cognition may be intensified by commercial behavior. The renowned surgeon Gawande [123] graphically portrayed that “In their [hospitals and the life sciences industry] subconscious, they view patients as mobile ATM machines.” The fundamental objective of commercial enterprises operating in the field of VR is to maximize profitability, prioritizing financial gains over the protection of patients’ rights. They seek to maximize the number of patients with chronic pain who undergo VR interventions for financial gain. In pursuit of this objective, some immoral VR commercial enterprises may resort to unscrupulous tactics to enhance the adoption or acquisition of VR technology, with the aim of generating substantial financial gains. For instance, tempted by the need to maximize profits, health care providers may engage in disease-creating (ie, defining the normal evolution that occurs during a human being’s birth, growth, strength, and old age as a disease or illness phenomenon and then taking measures to treat it; disease creation has become a commercial practice with the goal of “creating an artificial condition” rather than restoring the human body’s natural state) medical practices. This might disturb patients with chronic pain with regard to cognition of their own health conditions and coerce them into undergoing unnecessary VR interventions.

Moreover, developments such as eye tracking and emotion capture significantly enhance designers’ capacity to create addictive virtual “serious” games. Designers possess the ability to adapt and optimize virtual “serious” games in accordance with the preferences and emotional states of patients by using the data acquired from patients’ eye movements and facial changes. This guarantees that patients spend more time immersed in these virtual “serious” games [122]. Designers can deliberately design addictive mechanisms in VR in an attempt to keep patients with chronic pain uncontrollably immersed in painless VEs, allowing patients who believe that they have agency and control to become “addicted” to VR, making them dependent on VR. This phenomenon can be similar to the addictive mechanisms analyzed by Natasha Dow Schüll in her book titled *Addiction by Design: Machine Gambling in Las Vegas*. In the gambling industry, constant updates in surveillance technology serve the purpose of maintaining a perpetual state of immersive harmony between gamblers and machines. This immersive experience enables individuals to temporarily escape their worries and enter a “wonderland” while becoming mice within a “Skinner box.” Similarly, the commercialization of VR technology has the potential to incentivize designers to exploit patients’ autonomy by means of immoral VR designs. In the given context, VR interventions will easily shift from being initially centered on patients with chronic pain to being centered on capital profit or even power. Patients with chronic pain are turned from objects of medical care into controlled objects. Patients who are controlled by capital and power cannot possibly have a clear cognition of their own medical conditions, exacerbating their cognitive vulnerability.

Therefore, technicians have the responsibility to reflect about their standpoint on this issue and exercise prudence in the development and application of VR for manipulation, taking precautions to prevent any motivations rooted in avarice or other ambitions that extend beyond therapeutic intentions.

#### Privacy Leakage

Privacy plays a crucial role in safeguarding the essential conditions for moral personality or normative agency. Without a certain level of privacy, most individuals would not be comfortable in exploring certain ideas, expressing particular opinions, or acting in specific ways [122]. The more individual privacy is eroded, the more vulnerable individuals become to manipulation.

Regrettably, the emergence of VR technology may, in certain instances, aggravate privacy threats. Various entities, including hackers, malware, commercial businesses, governmental agencies, or criminal organizations, may be capable of violating privacy. These entities have the ability to use the privacy data they gather to manipulate individuals in specific ways and accomplish their desired goals strategically.

In contrast to how basic personal information is usually recorded in medical records for people with chronic pain, using VR interventions makes it possible to collect a wide range of individualized physical data by using motion capture technology, tracking technology, and other similar methods. For instance, HMDs commonly use advanced tracking technology, such as cameras and other positioning sensors, to track the patients and replicate their actions within the VE. Although these devices exert their intended technological function, they can also be used to surreptitiously monitor the patient and steal their personal data [124]. The collected physical information of patients may also encompass eye movement patterns, motor responses, and reflexes, which are uniquely linked to the individual’s identity, collectively constituting their distinctive “kinematic fingerprint” [119] In addition, more information pertaining to the habits and inclinations of patients could conceivably be collected, with the possibility for this information to be retained and used in ways that may threaten the privacy of individuals [119]. Apart from documenting patients’ explicit disclosures, technological devices have the capability to capture inadvertent manifestations that patients did not intend to reveal, including their unconscious physiological responses or subtle facial microexpressions. In the past, acquiring this information typically necessitated conspicuous and close observation or the engagement of experts. However, the integration of capture devices and VR technology has facilitated the collection of patients’ physical data, making it easy and more precise [122]. Documenting patients’ personal information often involves obtaining their informed consent before the implementation of a VR intervention. Nevertheless, it is often the case that patients lack a comprehensive understanding of the exact nature and extent of personal information that will be gathered throughout the VR intervention procedure. It is important to note that VR interventions for patients with chronic pain are not isolated events. Given the long-term nature of chronic pain therapy, VR interventions are conducted multiple times. The use of several VR interventions leads to the accumulation of a substantial volume of personal data and information within the system. This may encompass a wide range of information, spanning from the patient’s physical attributes to facts about their actions, emotions, and psychological state and their location and surrounding environment [119]. When the physical and mental states of a patient and their patterns of movement are frequently recorded, it becomes much easy to manipulate them. Hence, it is imperative for technological developers to possess in-depth awareness of the sensitivity of these data and the potential hazards associated with their improper use.

Currently, the use of VR technology as an intervention for chronic pain remains in the research stage. Hence, the transmission of data at a distance, which is necessary for conducting such a study, becomes an integral aspect of the privacy issue. The remote transmission of data introduces yet another potential entry point for attacks on the privacy of patients. This is particularly concerning owing to the fact that the origin of the transmission may reveal further information about the patient’s identity, such as their IP or email address [125]. Moreover, with the increasing adoption of VR technology in chronic pain, there is growing probability of VR technology being integrated with the internet. This will lead to increased likelihood of leakage of patient privacy.

By exploring the ethical challenges of VR technology interventions on the vulnerability of patients with chronic pain, our analysis anticipates that the ethical concerns arising from the immersive and manipulative attributes of VR technology have the potential to threaten and challenge the inherent vulnerability and cognitive vulnerability of patients with chronic pain (Table 1).

**Table 1:** Vulnerabilities and ethical manifestations in patients with chronic pain undergoing virtual reality (VR) technology interventions.

Types of vulnerabilities	VR technical attributes	Ethical issues with examples
Inherent vulnerabilities	VR immersion	High degree of immersion: Causes actual damage to patients, such as motion sickness and potential harms of the physical environment Long-term immersion: VR addiction and derealization syndrome
Cognitive vulnerabilities	VR manipulation	False sense of agency: Violates patient autonomy; controls the information patients receive; false and biased representations have negative impacts Commercialization of VR technology: Disease-creating medical practices; designing a VR “addiction” mechanism Privacy leakage: A large amount of personal information, including behavior, emotions, psychological aspects, and habits, is recorded and stored

## Responsible VR Interventions: Follow the Principle of Vulnerability

The application of VR technology in chronic pain has been proven to effectively alleviate pain, making VR technology one of the methods to reduce the vulnerability of patients with chronic pain. However, technology not only helps mitigate vulnerabilities but also creates new risks, vulnerabilities, and potential for accidents [126]. In the preceding section, we discussed the potential of VR technology to induce new vulnerabilities in patients with chronic pain. These findings indicate that there are existing or potential ethical risks associated with VR technology that may further deteriorate the health conditions of patients. Confronted with disparities in power and material resources, technical developers are afforded increased agency and, correspondingly, are charged with the responsibility of safeguarding vulnerable patients. Hence, to ensure the conscientious implementation of VR technology within the domain of chronic pain management, we propose that technical personnel adhere to the principle of vulnerability during the development or use of VR systems for patients with chronic pain. At each phase of VR technology development and implementation, a meticulous focus is placed on the vulnerability of patients with chronic pain, and thorough deliberation is allocated to the technical response strategies that could potentially heighten the patients’ vulnerability stemming from VR interventions. This practice is essential for promptly adapting the design, innovation, and developmental trajectory of VR technology and is dictated by the ethical responsibilities of technical personnel toward vulnerable patients with chronic pain.

From a phenomenological perspective, the patient’s body should not only be seen as the object of treatment but also as the embodiment of the patient’s relationship with the world; their actions within it; and how this influence is experienced and shapes one’s emotions, perceptions, and behaviors. Therefore, the vulnerability of the patient’s body and their needs are considered to have an impact on emotions, perceptions, and knowledge [101]. Technicians should be cognizant of the fact that the vulnerability experienced by patients with chronic pain can extend beyond the physical dimension, potentially affecting various personal aspects and social interactions. Mostly, vulnerability is used as a specific rather than a general characteristic [126]. Similar to the observation that VR interventions are inclined to amplify the inherent vulnerability of patients with chronic pain more than those of healthy individuals, it becomes imperative for technicians to conscientiously craft VEs that align with the distinctive vulnerability profiles of patients with chronic pain, thereby adhering to ethical and moral imperatives.

We have explored how VR interventions can potentially increase the inherent vulnerability of patients with chronic pain and lead to the emergence of new cognitive vulnerabilities, which can negatively affect various aspects of an individual’s physical or psychological well-being. By conducting a detailed analysis of the factors and specific manifestations of patient vulnerability resulting from VR interventions, we aimed to propose the ethical recommendations consistent with the principle of vulnerability, with the gradual implementation of responsible VR interventions in mind (Textbox 1).

**Ethical recommendations for implementing responsible virtual reality (VR) interventions under the principle of vulnerability.**

**Recommendations**

- Following a comprehensive and profound examination of the causes contributing to the heightened vulnerability of patients with chronic pain, elucidate the precise responsibilities and ethical obligations that technical developers must assume in the design and innovation of VR technology tailored to the needs of patients with chronic pain.
- Establish a set of experience-based ethical standards for VR development and intervention, rooted in the vulnerability characteristics of patients with chronic pain, and embed them in both software and hardware. Ensure that the operation of virtual environments adheres to ethical guidelines, thereby resisting any malicious intentions from developers or stakeholders with irresponsible actions toward patients [127].
- VR intervention standards should not simply exclude vulnerable patients, such as older adults or individuals with epilepsy. Technical developers can consider solutions such as inclusive design to promote the fairness and generalizability of VR interventions for populations with chronic pain.
- Adopt a citizen science research model or further involve patients with chronic pain as members of VR technology development teams or as equal research partners. Emphasize the voices of particularly vulnerable patients within the population with chronic pain in the early stages of VR technology design and development. Pay special attention to translating the practical experiences of these patients into theoretical knowledge, eliminating personal, subjective biases and ability biases (creating solutions using our own abilities as a baseline, known as ability biases) among technology developers during VR design.
- Refrain from bestowing unwarranted paternalistic protection upon patients with chronic pain, given their elevated vulnerability. Paramount importance should be accorded to the encouragement of patient autonomy and self-determination via VR interventions, thereby empowering patients to proactively manage their pain by exercising internal control, as opposed to acquiescing to passive acceptance of medical behavioral interventions.

To facilitate the ethical advancement of VR technology for the treatment of chronic pain and to address the ethical intricacies associated with patient vulnerability, it is imperative to infuse a comprehension of vulnerability across the entirety of the VR technology’s life cycle, spanning design, application, and dissemination. During the phase of technical design, it is of paramount importance to incorporate a profound consideration for the vulnerability of patients with chronic pain as a fundamental conceptual underpinning, thus elevating the importance of safeguarding vulnerability to a central tenet in the design framework. The emphasis is placed on respecting the autonomy of patients with chronic pain and encouraging their active involvement in all stages of the technical design process.

In the realms of management, application, and technology dissemination, a dual focus on respecting privacy and accumulating pertinent data through sustained patient feedback is upheld. A dynamic ethical management system and mechanism are adopted to respond to the evolving ethical landscape. Given the multifaceted challenges that VR technology encounters in addressing vulnerability within the context of long-term chronic pain management, a comprehensive ethical strategy is underscored. This strategy seeks to ingrain the principles of vulnerability ethics throughout the complete life cycle of VR technology design and application.

## Conclusions

Our paper provides a comprehensive overview of the current methods and principles underpinning VR technology interventions for chronic pain management. VR, as an emerging modality for addressing chronic pain, possesses the inherent attributes of immersion and maneuverability. Technical developers leverage these attributes to use VR interventions for mitigating chronic pain in patients. Nevertheless, this approach may also introduce heightened vulnerabilities among patients with chronic pain. To validate our hypothesis, we undertook a prospective examination of the potential ethical challenges that VR technology interventions may pose to the vulnerabilities of patients with chronic pain. This examination explored the emergent categories of vulnerability that VR interventions may generate and the specific manifestations of augmented vulnerability in patients.

The research findings indicate that, under improper or intentional guidance from technology developers, VR interventions may exacerbate the inherent vulnerabilities of patients with chronic pain while promoting the development of new cognitive vulnerabilities. To fully harness VR technology for the management of chronic pain and effectively mitigate the ethical risks associated with VR interventions, the expeditious realization of responsible VR interventions is imperative.

Consequently, we advocate that technology developers follow the principle of vulnerability at each stage of VR technology development and innovation, ensuring utmost patient-centered focus and safeguarding patient well-being.

## Abbreviations

HMD: head-mounted display
VE: virtual environment
VR: virtual reality
